# Motor function tests as early indicators of cognitive and functional decline in older adults: A correlational study

**DOI:** 10.1371/journal.pone.0338646

**Published:** 2025-12-22

**Authors:** Jarosław Fugiel, Stanisław H. Czyż, Anna Rohan, Karolina Lindner, Izabela Winkel, Małgorzata Sobieszczańska

**Affiliations:** 1 Department of Biological Principles of Physical Activity, Wrocław University of Health and Sport Sciences, Wrocław, Poland; 2 Faculty of Physical Education and Sports, Wroclaw University of Health and Sport Sciences, Wrocław, Poland; 3 Faculty of Sport Studies, Masaryk University, Brno, Czechia; 4 Physical Activity, Sport and Recreation (PhASRec), Faculty of Health Sciences, North-West University, Potchefstroom, South Africa; 5 Morphology and Embryology Department, Wrocław Medical University, Wrocław, Poland; 6 Department of Physiology and Pathophysiology, Division of Pathophysiology, Wrocław Medical University, Wrocław, Poland; 7 Research and Education Dementia Center in Ścinawa, Ścinawa, Poland; 8 Department and Clinic of Geriatrics and Internal Medicine, Medical University in Wrocław, Wrocław, Poland; Instituto Nacional de Geriatria, MEXICO

## Abstract

**Objectives:**

This study aimed to explore the relationship between selected motor performance tests and indicators of cognitive impairment in older adults. Specifically, it examined associations with the Mini-Mental State Examination (MMSE) and the Lawton Instrumental Activities of Daily Living (IADL) scale. A secondary aim was to assess whether these motor tests correlate more strongly with cognitive and functional status than with chronological age.

**Methods:**

Sixty-two institutionalized adults aged 60 years and older were evaluated. Motor performance was measured using tasks from the Vienna Test System (MLS), including pin insertion, tapping, targeting, tremor control, and line tracking. Handgrip strength was also assessed as a reference. Pearson’s correlation coefficients were calculated to examine relationships among motor test scores, MMSE, IADL, and age.

**Results:**

Most fine motor tests showed moderate to strong correlations with MMSE and IADL scores, but not with chronological age. Pin insertion, tracking, and tremor control were especially indicative of cognitive and functional status. Handgrip strength, by contrast, was significantly associated with age but not with MMSE or IADL.

**Conclusion:**

Fine motor tasks, particularly pin insertion and tracking, show significant associations with cognitive and functional performance in older adults. These findings suggest that such tasks may hold potential as practical indicators for identifying individuals at risk of decline; however, longitudinal studies are needed to confirm their predictive value and causal relationships.

## Introduction

There is a well-documented global trend of increasing incidence and prevalence of neurodegenerative diseases among the elderly population, including Alzheimer’s disease (AD), and various forms of dementia [1,2]. These conditions not only affect individuals and their families but also impose a substantial and escalating burden on healthcare systems and national economies worldwide [3–5]. The growing prevalence underscores the urgent need for both preventive strategies and effective methods to mitigate their socioeconomic impact.

Timely intervention remains one of the most promising approaches to alleviating the long-term effects of neurodegenerative diseases [6]. However, such intervention is only possible when the disease is detected at its earliest possible stage. While several diagnostic and screening methods are available [7], there is increasing interest in the potential of motor function assessments as early indicators of cognitive decline.

Given the critical role of primary healthcare professionals in the early recognition and diagnosis of dementia [8], there is a pressing need for simple, inexpensive, and rapid assessment tools that can be easily integrated into routine evaluations. Such tools should also be accessible to non-specialists, including family members and caregivers, who often observe early functional changes in the elderly. Commonly used screening tools include tests for manual dexterity [9] or tremor [10]. Although a definitive causal relationship between motor dysfunction and diseases such as dementia and Parkinson’s disease has yet to be established, correlational evidence is steadily accumulating.

On the other hand, older adults typically exhibit significantly reduced cognitive and motor proficiency, including declines in fine motor skills [11], stepping [12], and visual tracking [13]. These changes are often considered part of the normal aging process, but they may also reflect the early stages of neurodegenerative conditions. Therefore, it is crucial to identify which specific motor proficiency tests could serve as reliable early indicators of diseases such as, Alzheimer’s disease (AD), or other forms of dementia. A key question is whether these motor tests correlate primarily with age—reflecting general aging processes—or whether they are more closely associated with cognitive decline, thus pointing to early pathological changes rather than typical senescence.

Therefore, the objective of this study was to investigate the relationship between various motor performance tests and indicators of cognitive decline and functionality impairments in the elderly, specifically using the Mini-Mental State Examination (MMSE), and the Lawton Instrumental Activities of Daily Living (IADL) scale. In addition, the study examined the extent to which these motor tests correlated with participants’ age, in order to distinguish age-related effects from cognitive and functional impairment.

The motor tests are widely used by physiotherapists, sports trainers, and psychologists, and due to their simplicity, they can also be employed by individuals within the elderly person’s social circle. They are cost-effective, require minimal equipment, and can be conducted without the need for specialized settings.

Additionally, we included the handgrip strength test as a reference measure. Previous studies suggest that handgrip strength may serve as a risk indicator for adverse cognitive outcomes, including cognitive impairment, dementia, and Alzheimer’s disease [14]. By comparing performance across these tests, we aim to contribute to the identification of practical and scalable screening strategies for early signs of neurodegeneration in the aging population.

## Method

This cross-sectional research was approved by The Ethics Committee of Wroclaw Medical University nr KB-364/2017. All participants provided written informed consent prior to participation. They were informed that their involvement was voluntary and that they could withdraw from the study at any time without providing a reason and without any negative consequences. The participants were recruited between 01^st^ June and 31^st^ July 2017.

### Participants

The study was conducted at the Research and Educational Center for Dementia Diseases, Wroclaw Medical University, Poland. From all individuals residing at the center, those meeting the inclusion criteria were selected. The participants were not residents of a long-term care institution; they were older adults diagnosed with mild cognitive impairment or mild-stage dementia (mainly of the Alzheimer’s type) during a short-term, 2–4-week diagnostic stay at the Center for Dementia Diseases, after which they returned home and continued their usual daily activities.

The inclusion criteria for the study were: age > 60 years, without dementia or diagnosed as mild dementia (according to MMSE scores: 26–24 indicates mild cognitive impairment and 19–23 points indicates mild dementia), under institutional care, absence of other health contraindications for motor tests as determined by a physician, consent to participate in the study, and the ability to understand instructions and tasks. Participants were informed about the nature of the study and understood the exercise procedures and they went through a medical check and qualification (by the neurologist, psychiatrist, and psychologist).

Out of 81 individuals qualified for the study, 4 did not consent to participate, and 2 withdrew without providing a reason during the study. Consequently, 75 individuals were participating, (29 men and 46 women). The mean age of males with cognitive impairments was 72.9 ± 7.8 years, and females with cognitive impairments 77.2 ± 8.0 years.

### Instruments

1. To assess hand motor skills, tests from the MLS battery included in the Vienna Test System (Dr G. Schuhfried GmbH, Mödling 1986–1999) which is a reliable and objective tool to measure psychological constructs [15], were utilized. The MLS, (German: Mehrfachbelastung Labyrinth Sortieren means Multitasking – Labyrinth, Sorting) battery evaluates fine motor performance (finger, hand and arm movement) on a test board with various contact surfaces where participants perform a series of static and dynamic tasks using a contact pencil. The MLS is a modular test based on Fleishman’s factor analysis of fine motor skills.

In this study, we selected five subtests from the Motor Performance Series (MLS) of the Vienna Test System, i.e., tremor, tracking, aiming, tapping, and pin insertion, to comprehensively assess fine motor performance – manual dexterity. These tasks reflect different aspects of manual dexterity, including precision, coordination, steadiness, and psychomotor speed. Manual dexterity is a specific component of the fine motor performance and represents “*precise, diverse and flexible behavior that involves the coordination of many segments and whose repertoire can be expanded through learning.*” [16] (p.741).

The S3 short form of the MLS tests, as adapted by Vasella, was administered. The tests encompassed fine motor skills to assess rapid and precise hand movements:

- Tremor Test: This test determines the extent of tremor in the right and left hands by attempting to keep the hand steady with the pencil placed in a hole. Performance was evaluated based on the number of errors [n], defined as the pencil touching the wall or bottom of the hole. Participants placed the pencil in a 5.8 mm diameter hole, ensuring the pencil did not touch the sides or bottom. This position had to be maintained for 32 seconds (n/32s).
- Tapping Test: This test assesses the speed of movements in the right and left hands by counting the number of taps on a 40 x 40 mm sensor with the pencil. Movements were performed as quickly as possible within 32 seconds [n/32s].
- Pin Insertion Test: This test evaluates the speed of finger movements and hand-eye coordination in the right and left hands by measuring the time [s] taken to transfer 25 pins one by one from an initial position in a box 30 cm away to appropriate holes on the workboard from top to bottom, using the right or left hand accordingly.
- Targeting Test: This test measures the speed and accuracy of right- and left-hand movements by recording the time [s] to touch 20 sensors (5 mm in diameter and 4 mm apart) arranged vertically on the panel, and the number and duration of errors [n], defined as touches outside the sensor. Movements are performed from right to left for the right hand and in the opposite direction for the left hand.
- Line Tracking Test: This test assesses the speed of right and left hand movements with directional changes by measuring the time [s] taken to move a pencil placed in a groove with a variable shape from the start to the endpoint, and the number of errors [n], defined as touching the pencil to the groove walls. The task is performed with the right and left hands towards the left side of the workboard.

The tests were conducted using both the dominant and non-dominant upper limbs, as identified by the participants before the measurements began.

1. To assess cognitive impairment The Mini-Mental State Examination (MMSE), also referred to as the Folstein test, was administrated [17]. The MMSE aids in estimating the severity and progression of cognitive impairment and tracks cognitive changes in individuals over time, making it a reliable and valid [18] tool for documenting an individual’s response to treatment.

The MMSE is a 30-point questionnaire commonly used in clinical and research settings for dementia screening. It is divided into two parts. The first section assesses orientation, attention, and memory, with a maximum score of 21 points. The second section evaluates the ability to name objects, follow verbal and written instructions, read, write, and copy a figure, with a maximum score of 9 points. Scores range from 0 to 30, with higher scores indicating better cognitive function. A score of 24 or above is generally considered normal. Scores below 24 may suggest cognitive impairment and are often categorized as follows:

- Mild impairment: 18–23
- Moderate impairment: 10–17
- Severe impairment: 0–9

1. The Lawton Instrumental Activities of Daily Living (IADL) Scale [19] was used to assess functional status of the participants. Participants’ independent living skills, i.e., how a person is functioning at the present time, were evaluated.

IADL is a reliable [20] tool for assessing functioning, planning care and maintaining progress for elderly people with dementia.

The scale measures eight domains of independent living skills:

- Ability to use the telephone
- Shopping
- Food preparation
- Housekeeping
- Laundry
- Mode of transportation
- Responsibility for own medications
- Ability to handle finances

Each domain is scored based on the level of independence. Scores typically range from 0 to 8. A higher score indicates greater independence. Interpretation of the score is as follows: 8 points – full independence; lower scores: increasing levels of dependence in daily instrumental activities.

1. Handgrip test – The dominant hand grip strength [kg] was evaluated using a Jamar 5030J1 SAEHAN hydraulic dynamometer.

### Procedure

The procedures were performed according to a fixed and identical protocol for all participants.

Participants selected for the project were referred to a team made up of physicians and a psychologist for assessment and qualification. All patients signed an informed consent form before taking part in the study. On the day of testing, qualified participants were brought in one at a time for measurements. The tests were conducted in a separate room during morning hours, with each session done individually.

Before starting the tests, each person was given clear instructions on how to perform the tasks and asked whether they understood the directions and were aware they could withdraw from the study at any time.

For the motor tests, participants first performed a trial run to get familiar with the tasks. Special attention was paid to the position of the tested hand—it was not allowed to rest on the table or on the device surface. The contact pen had to be held just like a regular pen or pencil.

### Statistical analysis

Descriptive statistics were calculated for all relevant variables, including means, standard deviations, and the 25^th^, 50^th^ (median), and 75^th^ percentiles.

The MMSE results were corrected for age and education level according to the formula and normative tables proposed by Mungas et al. [21] and recommended by the Interdyscyplinarna Grupa Ekspertów Rozpoznawania i Leczenia Otępień – Interdisciplinary Group of Experts for the Diagnosis and Treatment of Dementias [22].

Given the lack of established evidence for a causal relationship between motor function performance and cognitive impairments in the elderly, we employed a correlational analysis. Pearson’s correlation coefficient (r) was used to assess the strength and direction of linear relationships between motor and cognitive variables. Statistical significance was assessed at three thresholds: p < 0.05, p < 0.01, and p < 0.001.

All analyses were conducted using JASP (ver. 0.17.1).

## Results

The descriptive statistics for the participants are detailed in Table 1. Out of the 75 participants tested, there were instances of missing data, the most notably regarding *IADL* score (data form 25 participants were missing). Missing IADL data were not random but occurred among participants who had been staying in the Center for an extended period and therefore had limited opportunities to perform the everyday activities assessed by the IADL scale, which primarily reflects functioning in a community setting.

**Table 1:** Descriptive statistics for the participants: number of observations (valid and missing), means, standard deviations, minimal and maximal values, as well as medians (50^th^ percentile) and 25^th^ and 75^th^ percentiles.

	Valid (n)	Missing (n)	Mean	Std. Deviation	Min	Max	25^th^ percentile	50^th^ percentile	75^th^ percentile
**Age (years)**	75	0	75.151	7.444	57.300	88.200	69.400	76.110	81.100
**Height (cm)**	66	9	160.224	9.205	143.500	181.000	152.775	159.000	167.475
**Mass (kg)**	66	9	71.462	13.501	41.700	107.900	62.200	70.000	80.725
**BMI [kg/m2]**	66	9	27.809	4.662	17.810	37.140	24.360	26.550	31.137
**Hand grip RH (kg)**	75	0	26.400	11.706	5.000	59.000	18.000	24.000	35.500
**Hand grip LH (kg)**	75	0	24.520	11.342	5.000	50.000	17.500	22.000	33.000
**IADL**	50	25	19.240	4.740	10.000	27.000	15.000	20.000	22.750
**MMSE**	75	0	22.933	4.536	12.000	30.000	20.000	24.000	27.000

### Correlations between Age, IADL, and MMSE

Firstly, we calculated the correlations between *Age*, *IADL* score, and *MMSE* score and *Hand grip* strength (for left and right hand) (Table 2). We found significant and moderate positive correlation between *MMSE* and *IADL* tests results. Negative, moderate and statistically significant correlation was found between *Age* and *IADL* results. However, negative, negligible and non-significant correlation was found between *Age* and *MMSE* results.

**Table 2:** Correlations between *Age*, *IADL*, and *MMSE.*

Variable		Hand grip RH	Hand grip LH	Age	IADL	MMSE
**1. Hand grip RH**	Pearson’s r	—				
	p-value					
**2. Hand grip LH**	Pearson’s r	0.913***	—			
	p-value					
**3. Age**	Pearson’s r	−0.423***	−0.444***	—		
	p-value					
**4. IADL**	Pearson’s r	0.211	0.212	−0.393	—	
	p-value					
**5. MMSE**	Pearson’s r	0.168	0.225	−0.041	0.481***	—
	p-value					—

* p < .05, ** p < .01, *** p < .001.

Negative, moderate, and statistically significant correlations were found between *Hand grip* strength (both hands) and *Age*.

### Correlations between Age, IADL, MMSE, and tremor, tracking, aiming, tapping, pin insertion

**Fig 1:**
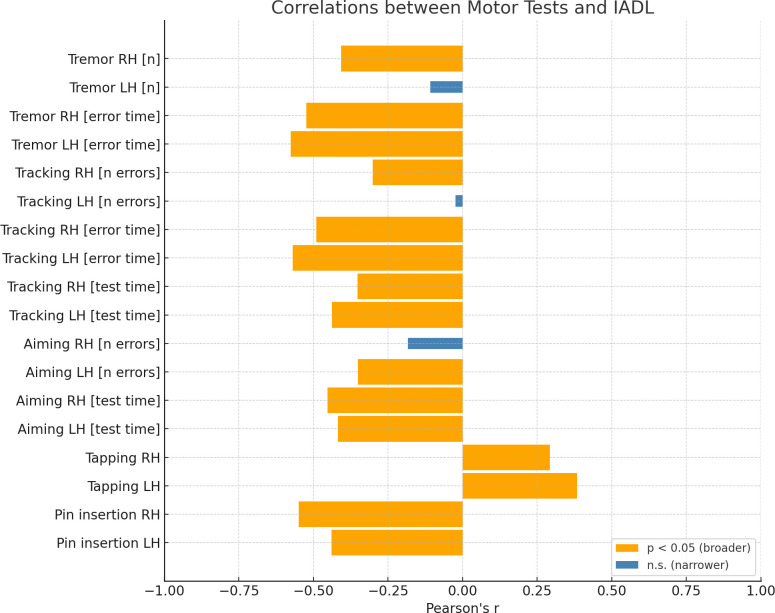
Correlations between motor test performance and Instrumental Activities of Daily Living (IADL) scores. Bar graph showing Pearson’s correlation coefficients (r) for each motor test in relation to IADL scores. Broader orange bars indicate statistically significant associations (p < 0.05), and narrower blue bars indicate non-significant results. RH = right hand; LH = left hand.

**Fig 2:**
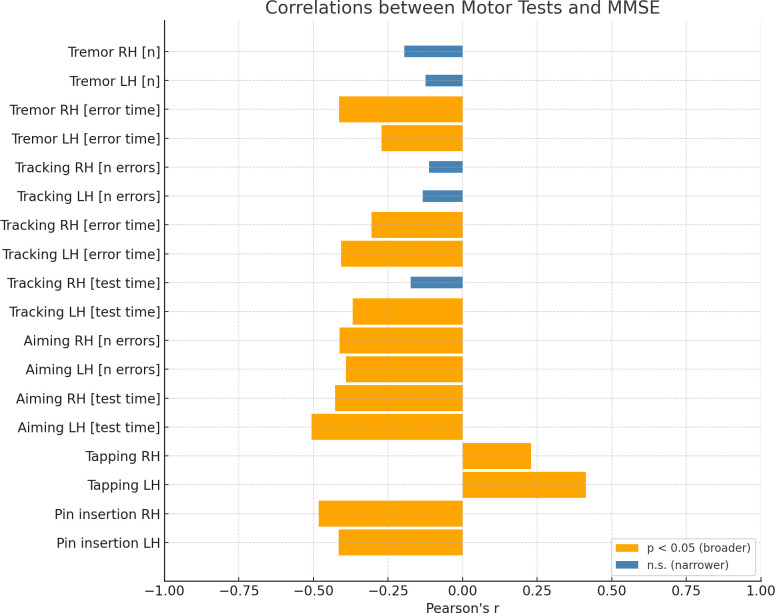
Correlations between motor test performance and Mini-Mental State Examination (MMSE) scores. Bar graph illustrating Pearson’s correlation coefficients (*r*) between each motor performance measure and MMSE results. Statistically significant correlations (*p* < 0.05) are shown as broader orange bars, while non-significant correlations (*p* ≥ 0.05) are represented by narrower blue bars. RH = right hand; LH = left hand.

Secondly, we calculated correlations between *Tremor*, *Tracking*, *Aiming*, *Tapping*, *Pin insertion* and *MMSE* score, *IADL* score and *Age* (see Table 3 for details). As it can be noticed, out of 18 variables included in the analyses, 15 and 13 variables related to manual dexterity turned out to be significantly correlated with *IADL* (Fig 1) and *MMSE* (Fig 2) scores (respectively). In most of the cases, these correlations were moderate and strong.

**Table 3:** Pearson’s r and p-values derived from pairwise correlation analyses.

Variable 1	Variable 2	Pearson’s r	p	Variable 1	Variable 2	Pearson’s r	p	Variable 1	Variable 2	Pearson’s r	p
**IADL**	Tremor RH [n]	−0.408	0.003	MMSE	Tremor RH [n]	−0.195	0.093	Age	Tremor RH [n]	0.17	0.144
**IADL**	Tremor RH [error time]	−0.524	< .001	MMSE	Tremor RH [error time]	−0.414	< .001	Age	Tremor RH [error time]	0.194	0.096
**IADL**	Tracking RH [n errors]	−0.301	0.034	MMSE	Tracking RH [n errors]	−0.113	0.333	Age	Tracking RH [n errors]	0.013	0.911
**IADL**	Tracking RH [error time]	−0.490	< .001	MMSE	Tracking RH [error time]	−0.306	0.008	Age	Tracking RH [error time]	0.124	0.289
**IADL**	Tracking RH [test time]	−0.352	0.012	MMSE	Tracking RH [test time]	−0.174	0.135	Age	Tracking RH [test time]	−0.121	0.299
**IADL**	Aiming RH [n errors]	−0.184	0.201	MMSE	Aiming RH [n errors]	−0.413	< .001	Age	Aiming RH [n errors]	0.067	0.566
**IADL**	Aiming RH [test time]	−0.453	< .001	MMSE	Aiming RH [test time]	−0.427	< .001	Age	Aiming RH [test time]	0.13	0.265
**IADL**	Tapping RH	0.292	0.04	MMSE	Tapping RH	0.23	0.047	Age	Tapping RH	−0.183	0.116
**IADL**	Pin insertion RH	−0.55	< .001	MMSE	Pin insertion RH	−0.482	< .001	Age	Pin insertion RH	0.306	0.008
**IADL**	Tremor LH [n]	−0.108	0.455	MMSE	Tremor LH [n]	−0.125	0.284	Age	Tremor LH [n]	0.002	0.986
**IADL**	Tremor LH [error time]	−0.576	< .001	MMSE	Tremor LH [error time]	−0.272	0.018	Age	Tremor LH [error time]	0.329	0.004
**IADL**	Tracking LH [n errors]	−0.024	0.87	MMSE	Tracking LH [n errors]	−0.134	0.25	Age	Tracking LH [n errors]	0.033	0.779
**IADL**	Tracking LH [error time]	−0.569	< .001	MMSE	Tracking LH [error time]	−0.408	< .001	Age	Tracking LH [error time]	0.165	0.158
**IADL**	Tracking LH [test time]	−0.438	0.001	MMSE	Tracking LH [test time]	−0.369	0.001	Age	Tracking LH [test time]	−0.019	0.874
**IADL**	Aiming LH [n errors]	−0.351	0.012	MMSE	Aiming LH [n errors]	−0.391	< .001	Age	Aiming LH [n errors]	0.011	0.923
**IADL**	Aiming LH [test time]	−0.418	0.003	MMSE	Aiming LH [test time]	−0.507	< .001	Age	Aiming LH [test time]	0.138	0.237
**IADL**	Tapping LH	0.383	0.006	MMSE	Tapping LH	0.413	< .001	Age	Tapping LH	−0.214	0.065
**IADL**	Pin insertion LH	−0.439	0.001	MMSE	Pin insertion LH	−0.416	< .001	Age	Pin insertion LH	0.252	0.03

RH – Right Hand.

LH – Left Hand.

**Fig 3:**
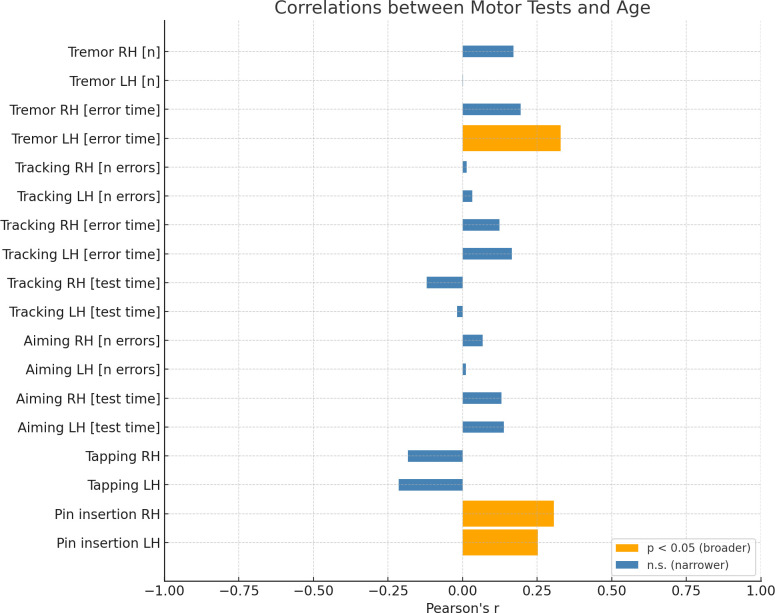
Correlations between motor test performance and age. Bar graph depicting Pearson’s correlation coefficients (r) between motor test results and participants’ age. Statistically significant correlations (p < 0.05) are displayed in orange, and non-significant correlations (p ≥ 0.05) in blue. RH = right hand; LH = left hand.

On the other hand, *Age* significantly correlated only with 3 variables out of 18 (Fig 3). Two of these variables were *Pin insertion* for the left and right hand (weak and moderate, appropriately). The third variable significantly correlated with age was *Tremor* left hand error time. The correlation was moderate.

## Discussion

The objective of this study was to investigate the relationship between various motor performance tests and indicators of cognitive decline in the elderly, specifically using the Mini-Mental State Examination (MMSE) and the functional decline using Lawton Instrumental Activities of Daily Living (IADL) scale. In addition, the study examined the extent to which these motor tests correlated with participants’ age, in order to distinguish age-related effects from cognitive impairment.

The results revealed several key findings. Most notably, IADL showed a moderate and statistically significant correlation with age, whereas MMSE did not. This is a particularly interesting observation, suggesting that IADL captures aspects of functioning that differ from those measured by the MMSE. As Sikkes et al. [23] noted, the interpretability of the Lawton IADL questionnaire for dementia patients is indeterminate. Therefore, IADL may measure older adults’ independence; however, this everyday functional independence does not solely depend on cognitive functioning. The MMSE may be a better indicator of cognitive impairment, which is not necessarily related to age. On the other hand, handgrip strength was negatively and moderately (significantly) correlated with age, but not with MMSE or IADL. This may be surprising, as cognitive decline is often associated with physical decline conspicuous in strength decline [14,24]. However, this could be due to the test we used, as Mitchell noted [25] that “*MMSE does not perform well as a confirmatory (case-finding) tool for dementia, mild cognitive impairment, and delirium, but performs adequately in a rule-out (screening) capacity*.” (p. 37).

Regarding our key findings, we observed that most motor function tests demonstrated significant moderate-to-strong correlations with both MMSE and IADL scores. Among the most robust findings were the correlations between the pin insertion test (for both the right and left hand) and both MMSE and IADL scores. These correlations were moderate to strong in magnitude, suggesting that poorer performance on pin insertion tasks is associated with lower cognitive functioning and diminished ability to perform daily activities. Similarly, the aiming tests showed moderate to strong correlations with both cognitive indicators (i.e., MMSE and IADL), indicating their sensitivity to cognitive performance. Notably, there was no correlation between aiming performance and chronological age. The observed inconsistencies between right- and left-hand associations may reflect underlying brain asymmetry [26] and cognitive deficits linked to structural and functional changes in the respective hemispheres, as described in age-related attenuation of dominant hand superiority [27].

An important observation was that in several cases, the motor tests correlated significantly with MMSE and IADL, but not with age. This distinction suggests that cognitive and functional decline may be more closely linked to changes in neural coordination and efficiency than to chronological aging alone. Such a pattern highlights the potential utility of these tests as early indicators of neurodegenerative processes rather than as mere reflections of age-related decline.The limited number of motor tests that correlated with age, primarily pin insertion and tremor error time for the left hand, were in the minority, reinforcing the notion that many of the selected tests may serve as specific indicators of cognitive and functional decline, independent of chronological aging.

### Limitations

Several limitations of the study should be acknowledged. First, the sample size was derived through convenience sampling from a single institutional care center, which may limit the generalizability of the findings to broader populations. Second, the cross-sectional design precludes any inference about the directionality or causality of the observed associations between motor performance and cognitive functioning. Third, although the motor tests used are validated and widely accessible, there may be inter-individual variability in test familiarity and effort that could influence results. Fourth, although the MMSE and IADL are well-established and widely used measures, both have inherent limitations: MMSE may show ceiling effects, particularly among individuals with higher cognitive functioning [28], while IADL performance can be influenced by cultural and contextual factors that affect the interpretation of independence in daily activities [29].

Finally, the study did not control for potential confounding factors such as comorbidities, medication use, or education level, which could have impacted both motor and cognitive performance.

## Conclusion

In conclusion, the study provides evidence that specific motor performance tests, particularly those assessing tremor and fine motor speed and accuracy, are significantly associated with cognitive functioning and daily living skills in older adults.

The absence of correlation with age in many cases supports their potential value as early indicators of functional and cognitive decline rather than age alone.

These findings are particularly relevant for primary healthcare professionals who are often the first point of contact for elderly patients. The simplicity, affordability, and low technological demand of these motor assessments, such as pin insertion, tapping, aiming, and tracking, make them ideal candidates for screening tools in community and clinical settings. Nevertheless, longitudinal research is needed to validate their predictive utility and clarify causal relationships.

## Supporting information

S1 File Raw data coded. (XLSX)
